# Trends in enforcement of National Comprehensive Cancer Network financial conflict of interest policy

**DOI:** 10.1093/jncics/pkae120

**Published:** 2024-11-26

**Authors:** Niloufar Saririan, Dedipya Bhamidipati, Pranam Dey, Sonia Persaud, Nirjhar Chakraborty, Sara Tabatabai, Grace Gallagher, Niti U Trivedi, Aaron P Mitchell

**Affiliations:** University of Florida, Gainesville, FL 32611, United States; Downstate School of Medicine, State University of New York, New York, NY 11203, United States; Department of Surgery, Brigham and Women’s Hospital, Harvard Medical School, Boston, MA 02115, United States; Department of Epidemiology and Biostatistics, Memorial Sloan Kettering Cancer Center, New York, NY 10017, United States; Department of Epidemiology and Biostatistics, Memorial Sloan Kettering Cancer Center, New York, NY 10017, United States; Department of Health Care Programs, NORC at the University of Chicago, Chicago, IL 60603, United States; Department of Epidemiology and Biostatistics, Memorial Sloan Kettering Cancer Center, New York, NY 10017, United States; Delfi Diagnostics, Baltimore, MD 21224, United States; Department of Epidemiology and Biostatistics, Memorial Sloan Kettering Cancer Center, New York, NY 10017, United States; Department of Medicine, Memorial Sloan Kettering Cancer Center, New York, NY 10065, USA

## Abstract

**Background:**

The National Comprehensive Cancer Network (NCCN) financial conflict of interest (FCOI) policy sets dollar maximums for panelists, but violations may occur.

**Methods:**

We studied NCCN Guidelines panelists for the 20 most prevalent cancers, 2013-2022. We included panelists with at least 1 full calendar year of service (“current panelists”) and those who began service during the study period (“new panelists”); NCCN FCOI policy limits ($20 000 from any single company or $50 000 across all companies) apply to both groups. Industry payments were obtained from Open Payments and mapped manually via National Provider Identifier. We calculated industry payments received, excluding the same payment categories as does NCCN (research, meals, travel and lodging). We estimated whether panelists received payments exceeding NCCN limits (“violation”). As a proxy for whether panelists were subsequently disqualified as stipulated, we measured continued service for at least 1 full calendar year (“retention”) subsequent to an estimated violation. We analyzed retention before and after 2016, due to increased scrutiny on NCCN FCOI in 2016.

**Results:**

The annual proportion of current panelists with estimated violations ranged between 0.5% (2020) and 5.8% (2016). Among panelists who did vs did not have violations, retention was 83.6% vs 88.5% during 2014-2015 (odds ratio [OR] = 0.55, 95% CI = 0.26 to 1.31) and 46.6% vs 89.4% during 2017-2020 (OR = 0.10, 95% CI = 0.06 to 0.17). Among new panelists, 2.7% (5/185) had prior-year violations during 2014-2015, as did 5.5% (18/330) during 2017-2021.

**Conclusions:**

Each year, a small portion of panelists receive industry payments exceeding NCCN limits. Since 2016, the likelihood that such panelists will continue to serve has decreased substantially.

## Introduction

Clinical practice guidelines play a critical role in health-care delivery, defining clinical standards-of-care and determining coverage and reimbursement policy by public and private payers. That these guidelines remain objective and free of commercial influence is therefore of high importance.^1^^,^^2^

The National Comprehensive Cancer Network (NCCN) Guidelines are the most widely used set of clinical practice guidelines within oncology. The NCCN maintains a large number of Guidelines, one for each cancer site. Each Guideline is maintained by an expert panel (constituted largely but not exclusively by physicians), which meets regularly to update the Guideline with new evidence and practice standards. In order to minimize bias, the NCCN maintains a conflict-of-interest policy stipulating the maximum permissible financial conflict of interest (FCOI) among Guidelines panelists.^3^ Since 2015, the limits have been $20 000 per year for any one company and $50 000 per year across all companies; the NCCN includes some forms of industry FCOI (such as consulting, speaking fees, and ownership interests) in these totals but not others (such as research funding and free meals). Panelists exceeding these limits should be asked to resign their positions, and prospective panelists should not be asked to join.

Since its first data publication in 2014, the Open Payments system has made public all financial transactions between US physicians and drug and device manufacturers.^4^ In 2016, research assessing FCOI among NCCN panelists was first reported and then published,^5^^,^^6^ finding that a small number of panelists held FCOI in apparent violation of NCCN’s self-imposed limits. After this report, NCCN leadership restated its commitment to transparent disclosure and ongoing monitoring of panelist FCOI, including through the use of the newly available Open Payments tool. The NCCN uses Open Payments in “an annual analysis, conducted internally”^7^ and follows this with “direct queries to panel members” to determine whether a violation of NCCN FCOI policy truly occurred.^8^

The goal of this study was to assess trends in panelist FCOI and NCCN enforcement of FCOI policy since 2016.

## Methods

The names and affiliations of NCCN Guidelines panelists were obtained by manual review of all NCCN Guidelines documents for the 20 most common cancer sites published from 2013 to 2022. The National Provider Identifier (NPI) for each panelist was determined through a manual search of National Plan and Provider Enumeration System (NPPES) data. Nonphysician panelists and those without an identifiable NPI were excluded.

Panelists were mapped to their Open Payments records of industry payments by NPI (and by Open Payments profile ID for archival payments occurring in 2014). Our observation period for industry payments was 2014-2021, and we observed NCCN panel membership for an additional calendar year (through the end of 2022). For physicians who participated on multiple panels, each physician–panel pair was treated as a separate observation.

To align our study with NCCN policy, we focused on the payment types that the NCCN considers in its FCOI limits. Specifically, we included only ownership interests and “general payments” (eg, non-research related payments), and not the general payments subcategories of travel, lodging, and meals, which the NCCN explicitly excludes.^3^ Ownership interests contains two data fields to report the value of investment, “amount invested” and “value of interest”; in cases where these two values were reported as different, we used the smallest value.

We defined each physician’s term of service on a guideline panel as starting the day *after* the publication of the most recent guideline on which they were *not* a listed panelist, and continuing through the date of publication of the final/most recent guideline on which they were a listed panelist. If an individual had an otherwise continuous period of service as a panelist that was interrupted by a single guideline update on which they were not a listed panelist (but were listed again on the subsequent update), we assumed this reflected a temporary absence from an NCCN panel meeting and treated this as an unbroken period of service. If an individual had periods of service on a given panel that were separated by more than one guideline update on which they were not a listed panelist, we assumed these represented separate periods of service. In such cases, we included only the first time period in our analysis.

We analyzed industry payments within two overlapping groups of panelists: those who began service on a Guidelines panel during the observation period (“new panelists”), and those with one or more calendar years of service during the observation period (“current panelists”). Physicians could be included in both analyses if they both began service during the observation period and continued for at least 1 year thereafter. Among new panelists, to assess adherence to the NCCN policy that physicians with excessive conflicts not be selected for service, we analyzed industry payments received during the year before their appointment; we included those beginning service on January 1, 2015 or later to ensure one full year of observed, pre-appointment Open Payments data. Among existing panelists, to assess adherence to the NCCN policy that panelists not accept industry payments in excess of limits, we assessed industry payments received during each full calendar year of service; for simplicity, we did not analyze payments received during partial years.

In all cases, we tabulated payments and determined whether payments in the included categories exceeded either $50 000 overall or $20 000 per company per year, and we termed such events “violations.” To make inferences about NCCN enforcement of its policy, each time a violation occurred we assessed whether the panelist in question remained active (was “retained”) on the panel for an additional full calendar year subsequent to the violation. Among panelists who were retained, we tabulated industry payments in the post-violation year and determined if another violation (“repeat violation”) occurred. All analyses were conducted using nominal dollars, because the NCCN FCOI policy limits did not change during the study period.

To evaluate whether NCCN’s enforcement of its policy changed after 2016, we compared retention rates before and afterwards. We used logistic regression models to determine whether the occurrence of a violation within the index year, the time period (pre- vs post-2016), and the magnitude of violation (whether the violation exceeded the $50 000 or $20 000 threshold by less than vs more than 20%) were associated with the likelihood of retention in the subsequent year. Using generalized linear models, we also assessed whether the occurrence of a violation was associated with the subsequent year’s monetary total. An alpha of 0.05 was considered statistically significant.

All analyses were conducted in R.

## Results

We identified 1448 physician–guideline pairs. After deduplication and removal of those with a deactivated or unidentifiable NPI, 1263 pairs comprising 1092 unique physicians remained (Figure S1). Across the full study period, these physicians received 106 682 individual industry payments; 10 were ownership interests, and the remainder were general payments. In total, 604 pairs were eligible for analysis as new panelists, and 978 as current panelists.

### Current panelist analysis

Among current panelist–guideline pairs, the mean dollar value of industry payments received across all categories ranged from $11 847 in 2017 to a low of $5958 in 2020 (Table 1). Mean value including only categories relevant to the NCCN limit ranged between $9253 (2017) and $5433 (2020). The greatest value of NCCN-relevant payments a panelist received during a single calendar year was $561 856, occurring in 2016. Results were similar when analyzing by unique panelists as opposed to panelist–guideline pairs (Table S1). The distribution of payment categories was very similar to those previously reported among NCCN Guidelines panelists,^9^ with food and beverage payments being the most numerous, and speaking and consulting fees having the greatest dollar value (not shown).

**Table 1.** Payments received by NCCN Guidelines panelists during full calendar years of participation. Unit of analysis is the physician–guideline pair (eg, NPIs can be counted more than once); “20 000” refers to the NCCN maximum of $20 000 per individual company per year, and “50 000” refers to the NCCN maximum of $50 000 across all companies per year.

Characteristic	2014, N = 483	2015, N = 499	2016, N = 507	2017, N = 531	2018, N = 528	2019, N = 521	2020, N = 554	2021, N = 552	Overall, N = 4175
Number of payments									
Mean	16	14	14	15	14	14	4	4	12
Median	6	4	5	4	4	5	1	1	3
Maximum	141	137	133	169	185	156	78	45	185
Value of payments, all categories (USD)									
Mean	10 391	10 783	11 514	11 847	10 824	11 548	5958	6682	9880
Median	1193	904	1148	1010	1006	1980	145	958	1030
Maximum	434 087	179 988	568 546	187 724	212 424	145 574	70 557	113 646	568 546
Value of payments, NCCN categories (USD)									
Mean	6175	7908	8800	9253	8369	9169	5433	6492	7687
Median	199	0	39	0	275	1315	0	150	0
Maximum	80 405	163 465	561 856	185 090	173 103	141 661	66 756	112 712	561 856
Violation type (%)									
20 000 Only	8 (1.7)	6 (1.2)	5 (1.0)	9 (1.7)	11 (2.1)	2 (0.4)	1 (0.2)	4 (0.7)	46 (1.1)
50 000 Only	0	9 (1.8)	8 (1.6)	4 (0.8)	0	6 (1.2)	1 (0.2)	3 (0.5)	31 (0.7)
Both types	7 (1.4)	12 (2.4)	6 (1.2)	16 (3.0)	7 (1.3)	15 (2.9)	1 (0.2)	2 (0.4)	66 (1.6)
None	468 (97)	472 (95)	488 (96)	502 (95)	510 (97)	498 (96)	551 (99)	543 (98)	4032 (97)

Abbreviations: NCCN = National Comprehensive Cancer Network; USD = United States dollar.

**Figure 1.**
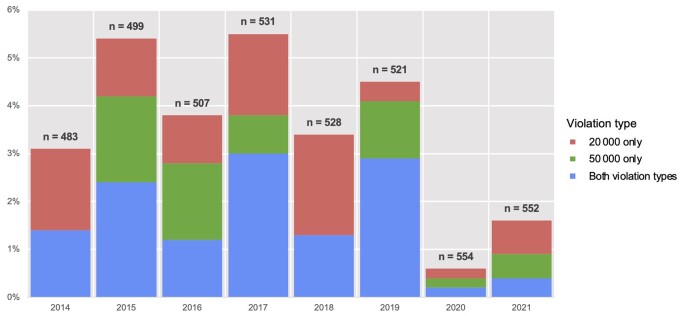
Panelists who received industry payments in excess of National Comprehensive Cancer Network (NCCN) Conflict of Interest policy, current panelist analysis. Y-axis represents the percentage of all eligible panelists who had violations in the indicated year. Unit of analysis is the physician–guideline pair (eg, National Provider Identifiers can be counted more than once); “20 000” refers to the NCCN maximum of $20 000 per individual company per year, and “50 000” refers to the NCCN maximum of $50 000 across all companies per year.

The percentage of panelist–guideline pairs with estimated violations of either the $20 000 single-company maximum, the $50 000 overall maximum, or both was between 3% and 6% of all panelists from 2014 to 2019 (Figure 1). A high of 29 (5%) panelist–guideline pairs had estimated violations in 2017. Violations appeared less frequently in 2020-2021, occurring among 3 pairs (0.5%) in 2020 and 9 pairs (1.6%) in 2021.

### New panelist analysis

Among new panelist–guideline pairs, the mean dollar value of industry payments received in the year before appointment across all categories ranged from a low of $3141 (2014) to a high of $14 325 (2017) (Table 2). Mean value including only categories relevant to the NCCN limit ranged between $1840 (2014) and $11 553 (2017). The greatest value of NCCN-relevant payments a panelist received in the year before joining was $214 656, occurring in 2015. Results were similar when analyzing by unique panelists (Table S2).

**Table 2.** Payments received by prospective National Comprehensive Cancer Network (NCCN) Guidelines panelists during the calendar year prior to the beginning of their participation on a panel. Unit of analysis is the physician–guideline pair (eg, National Provider Identifiers can be counted more than once); “20 000” refers to the NCCN maximum of $20 000 per individual company per year, and “50 000” refers to the NCCN maximum of $50 000 across all companies per year.

Characteristic	2014, N = 75	2015, N = 116	2016, N = 68	2017, N = 61	2018, N = 92	2019, N = 82	2020, N = 79	2021, N = 31	Overall, N = 604
Number of payments									
Mean	7	13	10	18	9	16	5	4	11
Median	3	2	3	7	2	6	0	2	2
Maximum	39	217	54	195	51	106	57	23	217
Value of payments, all categories (USD)									
Mean	3141	10 062	8138	14 325	6165	11 272	6377	4820	8236
Median	246	376	307	1732	262	1078	0	215	319
Maximum	42 246	289 266	52 944	183 386	59 500	95 421	96 512	81 950	289 266
Value of payments, NCCN categories (USD)									
Mean	1840	7618	6223	11 553	4572	8679	5608	4613	6404
Median	0	0	0	1100	0	0	0	0	0
Maximum	22 157	214 656	43 460	159 023	35 377	81 973	91 061	81 950	214 656
Violation type (%)									
20 000 Only	0	1 (0.9)	3 (4.4)	2 (3.3)	0	5 (6.1)	1 (1.3)	0	12 (2.0)
50 000 Only	0	0	0	1 (1.6)	0	0	0	0	1 (0.2)
Both types	0	5 (4.3)	0	3 (4.9)	0	3 (3.7)	2 (2.5)	1 (3.2)	14 (2.3)
None	75 (100)	110 (95)	65 (96)	55 (90)	92 (100)	74 (90)	76 (96)	30 (97)	577 (96)

Abbreviations: NCCN = National Comprehensive Cancer Network; USD = United States dollar.

**Figure 2.**
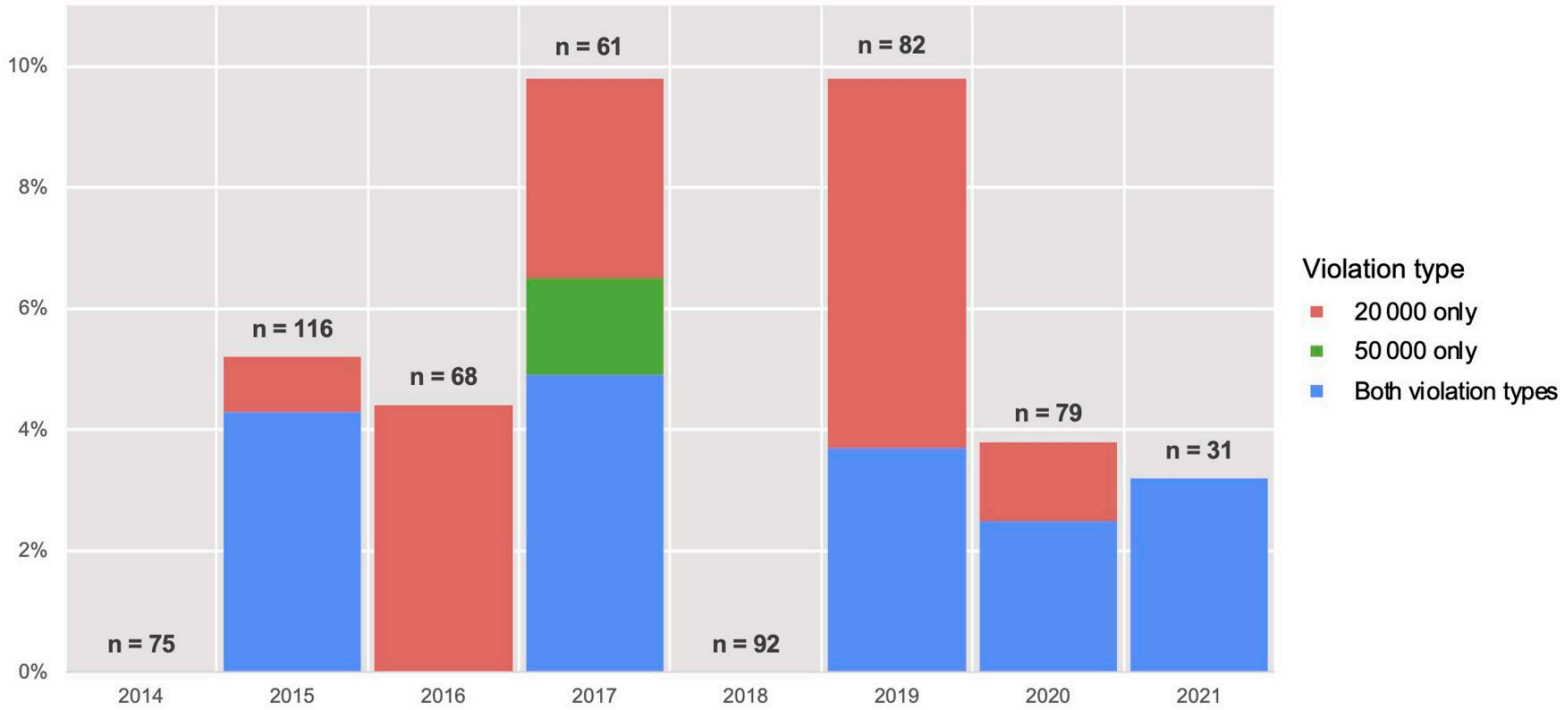
Panelists who received industry payments in excess of National Comprehensive Cancer Network (NCCN) Conflict of Interest policy, new panelist analysis. Y-axis represents the percentage of all eligible panelists who had violations in the indicated year. The year shown reflects the year prior to beginning participation; for example, violations occurring in 2016 reflect those by panelists who began participation in 2017. Unit of analysis is the physician–guideline pair (eg, NPIs can be counted more than once); “20 000” refers to the NCCN maximum of $20 000 per individual company per year, and “50 000” refers to the NCCN maximum of $50 000 across all companies per year.

In absolute terms, the year with the greatest number of new panelists with FCOI in excess of NCCN limits was 2020, with 8 panelists (8/82, 9.8%) having received excessive payments in 2019 (Figure 2). The greatest relative proportion occurred in 2018, with 6 of 61 (9.8%) having received excessive payments in 2017. In total, 2.7% (5/185) of those beginning service in 2014-2015 had estimated violations in the prior year, as did 5.5% (18/330) of those beginning in 2017-2021.

### Panelists receiving excessive payments

Compared with those who had not received excessive payments, panelists who received excessive payments continued to receive higher payment amounts compared with other panelists in subsequent years (Figure S2). Among panelists who received payments estimated to be in excess of NCCN’s policy during 2016, the median amount received by the same panelists in the subsequent year was greater than $60 000, well above the NCCN single-year limit.

**Figure 3.**
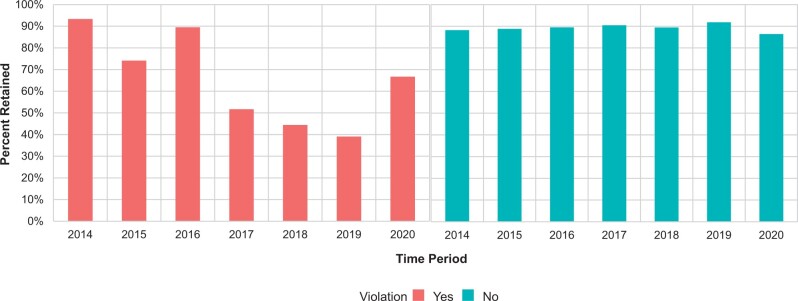
Proportion of NCCN Guidelines panelists retained, by presence of a violation. Unit of analysis is the physician–guideline pair (eg, NPIs can be counted more than once). The year shown is the index year, which is the year assessed for whether a violation occurred. Panelists were defined as “retained” if they continued service on the NCCN Guidelines panel for at least 1 additional year beyond the index year (eg, for 2017, retention for the duration of 2018 was assessed, and panelists were grouped with respect to whether a violation had occurred in 2017).

Among panelist–guideline pairs who did not have an estimated violation of NCCN FCOI limits, 89.2% were retained across the whole study period, ranging between 86.4% (2020) and 91.8% (2019) (Figure 3, Table S3). Panelist–guideline pairs with estimated FCOI violations were less likely to be retained; 63.4% were retained across the full study period (odds ratio [OR] = 0.21, 95% CI = 0.15 to 0.31). However, the likelihood of retention post-violation varied during the study period. During 2014-2015, 83.6% (34 of 42) were retained after estimated violation (vs no violation, OR = 0.55, 95% CI = 0.55 to 1.31), compared with 46.6% (34 of 73) during 2017-2020 (vs no violation, OR = 0.10, 95% CI = 0.06 to 0.17). The interaction of time period (2014-2015 vs 2017-2020) and estimated violation was statistically significant with *P* less than .001.

Panelists who had FCOI exceeding NCCN limits by 20% or more were less likely to be retained than those whose estimated violations did not exceed 20% (54.9% vs 76.9%, Fisher exact test *P* = .01) (Table S3).

## Discussion

In this study of NCCN FCOI policy, we observed a substantial change in policy enforcement during 2014-2022. Though a small number of NCCN Guidelines panelists continued to accept industry payments in excess of NCCN limits, the likelihood that panelists continued to serve after violations occurred decreased substantially.

We measured whether physicians continued to be listed as Guidelines panelists subsequent to a violation. This is inherently a proxy measure for whether panelists were directly asked to resign their seats in accordance with NCCN policy. However, several lines of evidence support that this is what was occurring in at least some of these cases. First, the turnover in panelists violating FCOI policy was substantially greater than in non-violating panelists (which was approximately 10%-15% per year) (Figure 3). Second, turnover was much more likely among panelists with large (>20%) violations than those who accepted industry payments “just above” the NCCN limit (Table S3). This could reflect discretion in policy enforcement or identification of a misattributed payment on NCCN’s manual review, which might bring a panelist back below the limit, which NCCN has claimed occurs regularly.^7^ Finally, we observed a large, statistically significant stepwise change in post-violation turnover after 2016, during which NCCN publicly restated its commitment to transparency and managing FCOI.^8^ Although this is an inference, the temporal association with the change in post-violation turnover would be highly coincidental.

An alternative explanation for this finding is that panelists begin to accept greater amounts from industry in anticipation of resigning from an NCCN panel. However, although this could explain the higher turnover among panelists with excessive FCOI, it would not explain the substantial change in turnover after 2016.

Although FCOI policy enforcement appears to have changed, it remains too soon to determine whether this has translated to panelist behavior. We observed a substantial decrease in the incidence of violations in 2020-2021 (Table 1), but this coincides with a well-documented broader decrease in industry payments to physicians.^10-12^ The pandemic-related decline in payments may or may not fully explain the decline in violations. Whether violations of NCCN FCOI policy increase to prepandemic levels as payments recover or remain lower potentially in response to enforcement remains to be seen.

Even as FCOI policy enforcement among current panelists appeared to increase, we did not observe any clear trend in the selection of new panelists with excessive FCOI (Figure 2). This may be an area for future emphasis as the NCCN seeks to manage FCOI, as prior research suggests there may be sufficient numbers of unconflicted potential panelists practicing at the same NCCN member institutions.^13^

This study is limited by the inclusion of the 20 most prevalent cancers. NCCN publishes Guidelines for more than 60 cancer types, and it is possible that payment patterns differ among panelists for lower-prevalence cancer types. We evaluated only those payment categories to which the NCCN applies its policy, notably excluding food and beverage, travel and lodging, and research payments. Food and travel are forms of value enjoyed by physician recipients, and research payments, although not received directly by physicians, may lead to professional benefits. Our method of analyzing only full calendar years of panel participation may bias downward our estimate of FCOI policy violations, as we would not capture any violations that occurred within the years that a panelist either joined or left the panel. Whether these forms of payment should be excluded when calculating physicians’ FCOI may therefore be subject to disagreement.

Most importantly, Open Payments data are not perfect. Although very accurate in aggregate,^14^^,^^15^ Open Payments is known to contain anecdotal instances of misattributed payments,^16-18^ which could be critical in cases of physicians near the NCCN payment threshold. The NCCN has previously stated that in its own (unpublished) review of Open Payments data, approximately half of apparent violations are due to “incorrect or misleading” reporting in Open Payments.^7^ This is potentially in line with our finding that, since 2017, only approximately one-half of panelists with excessive payments (per Open Payments data) continue to serve thereafter. If this were the same one-half for whom Open Payments data were incorrect in suggesting a violation, it would potentially imply that NCCN’s enforcement of its FCOI policy is near complete.

Establishing and enforcing an FCOI policy such as NCCN’s presents challenges and tradeoffs. Some panelists who exceeded NCCN limits received as much as $214 656 within a single calendar year; if members with this degree of industry involvement were to continue as panelists, it could result in the perception of conflict of interest and diminish trust in the guidelines. Indeed, payments of this value might have the true potential to sway opinion, consciously or subconsciously. An alternative policy approach to prioritize reducing FCOI could be apply stricter FCOI limits at the time of panelist nomination; prior work has identified a “pool” of oncologists at the same career stage and practicing at the same NCCN institutions—but with substantially lower FCOI—as those nominated to NCCN Guidelines panels.^13^ However, an FCOI policy based on a blanket dollar value limit risks excluding expert physicians whose industry interests are unrelated to the cancer type or therapies pertaining to their panelist role, and hence would not bias recommendations. An alternative policy approach to prioritize maintaining panel expertise while managing bias could be to remove blanket dollar thresholds while expanding the criteria that trigger panelist recusal from specific discussions; for example, current NCCN policy does not require the recusal of panelists who have received substantial research funding related to a drug under discussion.^3^

Receipt of industry money can negatively affect physician practice patterns^19-23^ and may also affect the recommendations they make in clinical practice guidelines.^24^ Close oversight and management of FCOI among the physician authors of clinical practice guidelines is therefore of high importance. Our findings suggest that NCCN has increased oversight and enforcement of its FCOI policy in recent years. However, new violations of this policy continue to occur, suggesting the potential for improved self-governance by physician panelists.

## Supplementary Material

pkae120_Supplementary_Data

## Data Availability

All data sources used in this study, including NCCN Clinical Practice Guidelines documents and Open Payments data, are already freely available to the public. A derivative dataset, the list of NCCN panelists for the Guidelines of all major cancer types from 2013 to 2024, is available to other investigators by request.
