# Supplementary material for: Trends in enforcement of National Comprehensive Cancer Network financial conflict of interest policy
Source: JNCI Cancer Spectr. 2024 Nov 26;8(6):pkae120. doi: 10.1093/jncics/pkae120 (PMC11671141; doi:10.1093/jncics/pkae120)
Supplement: pkae120_Supplementary_Data [file pkae120_supplementary_data.pdf]

Supplementary Material

Trends in Enforcement of National Comprehensive Cancer Network Financial Conflict of Interest Policy

Niloufar Saririan, BS,[1] Dedipya Bhamidipati, MBBS,[2] Pranam Dey, MD,[3] Sonia Persaud, MPH,[4] Nirjhar Chakraborty, MPH,[4] Sara Tabatabai, MA,[5] Grace Gallagher, MPH,[4] Niti U. Trivedi, MPH,[6] Aaron P. Mitchell, MD, MPH[4,7]

1. University of Florida, Gainesville, FL

2. State University of New York, Downstate School of Medicine, New York, NY

3. Yale University School of Medicine, New Haven, CT

4. Memorial Sloan Kettering Cancer Center, Department of Epidemiology and Biostatistics, New York, NY

5. NORC at the University of Chicago, Chicago, IL

6. Delfi Diagnostics, Baltimore, MD

7. Memorial Sloan Kettering Cancer Center, Department of Medicine, New York, NY

Table of Contents

	Page
Supplementary Figure 1: Selection of eligible National Comprehensive Cancer Network Guidelines panelists.	2
Supplementary Table 1. Payments received by unique physicians included in the "current panelist" analysis	3
Supplementary Table 2: Payments received by unique physicians included in the "new panelist" analysis	4
Supplementary Figure 2. Dollar value of payments received in subsequent year, grouped by whether an NCCN Conflict-of-Interest policy violation occurred in the index year	5
Supplementary Table 3. Likelihood of retention over time, by presence of a violation and magnitude of violation	6

Supplementary Figure 1: Selection of eligible National Comprehensive Cancer Network Guidelines panelists. Physician panelists on the Guidelines for the 20 most prevalent cancer types were manually abstracted from 2014-2022.

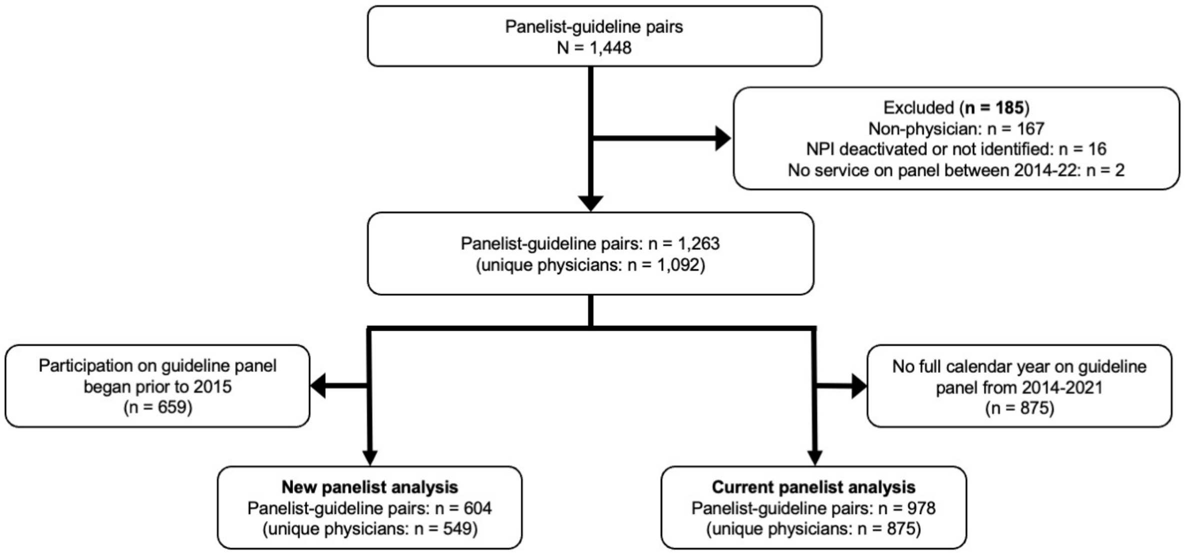

Supplementary Table 1. Payments received by unique physicians included in the "current panelist" analysis. Physicians' data are included only during years in which they were included in the "current physicians" analysis, but in this table are counted only once regardless of whether they served on one panel or multiple. The unit of analysis is the NPI-year. "20,000" refers to the NCCN maximum of $20,000 per individual company per year, and "50,000" refers to the NCCN maximum of $50,000 across all companies per year.

Characteristic	2014, N = 428	2015, N = 455	2016, N = 464	2017, N = 484	2018, N = 481	2019, N = 475	2020, N = 505	2021, N = 503	Overall, N = 3,795
**Number of Payments**									
Mean	15	13	13	14	14	14	4	5	11
Median	5	4	5	4	4	5	1	1	3
Maximum	141	137	133	169	185	156	78	45	185
**Value of Payments, All Categories (USD)**									
Mean	9,606	10,305	10,832	11,102	10,605	11,482	6,002	6,679	9,524
Median	1,115	904	1,020	940	1,050	1,974	171	500	987
Maximum	434,087	179,988	568,546	187,724	212,424	145,574	70,557	113,646	568,546
**Value of Payments, NCCN Categories (USD)**									
Mean	6,175	7,908	8,800	9,253	8,369	9,169	5,433	6,492	7,687
Median	152	0	33	0	310	1,100	0	0	0
Maximum	80,405	163,465	561,856	185,090	173,103	141,661	66,756	112,712	561,856
**Violation Type, N (%)**									
20,000 Only	7 (1.6)	5 (1.1)	5 (1.1)	8 (1.7)	11 (2.3)	2 (0.4)	1 (0.2)	4 (0.8)	43 (1.1)
50,000 Only	0	6 (1.3)	6 (1.3)	3 (0.6)	0	6 (1.3)	1 (0.2)	3 (0.6)	25 (0.7)
Both Types	5 (1.2)	11 (2.4)	5 (1.1)	13 (2.7)	7 (1.5)	13 (2.7)	1 (0.2)	2 (0.4)	57 (1.5)
None	416 (97)	433 (95)	448 (97)	460 (95)	463 (96)	454 (96)	502 (99)	494 (98)	3,670 (97)

Supplementary Table 2: Payments received by unique physicians included in the "new panelist" analysis. Physicians' data are included only during the year in which they were included in the "new panelist" analysis, but in this table are counted only once regardless of whether they served on one panel or multiple. The unit of analysis is the NPI-year. "20,000" refers to the NCCN maximum of $20,000 per individual company per year, and "50,000" refers to the NCCN maximum of $50,000 across all companies per year.

Characteristic	2014, N = 73	2015, N = 112	2016, N = 59	2017, N = 57	2018, N = 88	2019, N = 80	2020, N = 77	2021, N = 28	Overall, N = 574
**Number of Payments**									
Mean	7	12	10	19	9	16	5	4	11
Median	3	2	6	7	2	6	0	3	2
Maximum	39	217	54	195	51	106	57	23	217
**Value of Payments, All Categories (USD)**									
Mean	3,216	9,498	8,539	14,702	6,341	11,351	6,516	5,326	8,288
Median	246	316	771	1,350	280	1,078	0	242	339
Maximum	42,246	289,266	52,944	183,386	59,500	95,421	96,512	81,950	289,266
**Value of Payments, NCCN Categories (USD)**									
Mean	1,890	7,177	6,516	11,972	4,705	8,750	5,727	5,107	6,458
Median	0	0	0	1,000	0	0	0	0	0
Maximum	22,157	214,656	43,460	159,023	35,377	81,973	91,061	81,950	214,656
**Violation Type, N (%)**									
20,000 Only	0	1 (0.9)	2 (3.4)	2 (3.5)	0	5 (6.3)	1 (1.3)	0	11 (1.9)
50,000 Only	0	0	0	1 (1.8)	0	0	0	0	1 (0.2)
Both Types	0	4 (3.6)	0	3 (5.3)	0	3 (3.8)	2 (2.6)	1 (3.6)	13 (2.3)
None	73 (100)	107 (96)	57 (97)	51 (89)	88 (100)	72 (90)	74 (96)	27 (96)	549 (96)

Supplementary Figure 2. Dollar value of payments received in subsequent year, grouped by whether an NCCN Conflict-of-Interest policy violation occurred in the index year. Unit of analysis is the physician-guideline pair (eg., NPIs can be counted more than once). The year shown is the index year, which is the year assessed for whether a violation occurred; the payment totals represent payments received in the subsequent year (eg., for index year 2017, the value of 2018 payments are shown, grouped with respect to whether a violation occurred in 2017). Y-axis in USD. Note: 6 outliers with payments ranging $125,000-$561,856 are omitted.

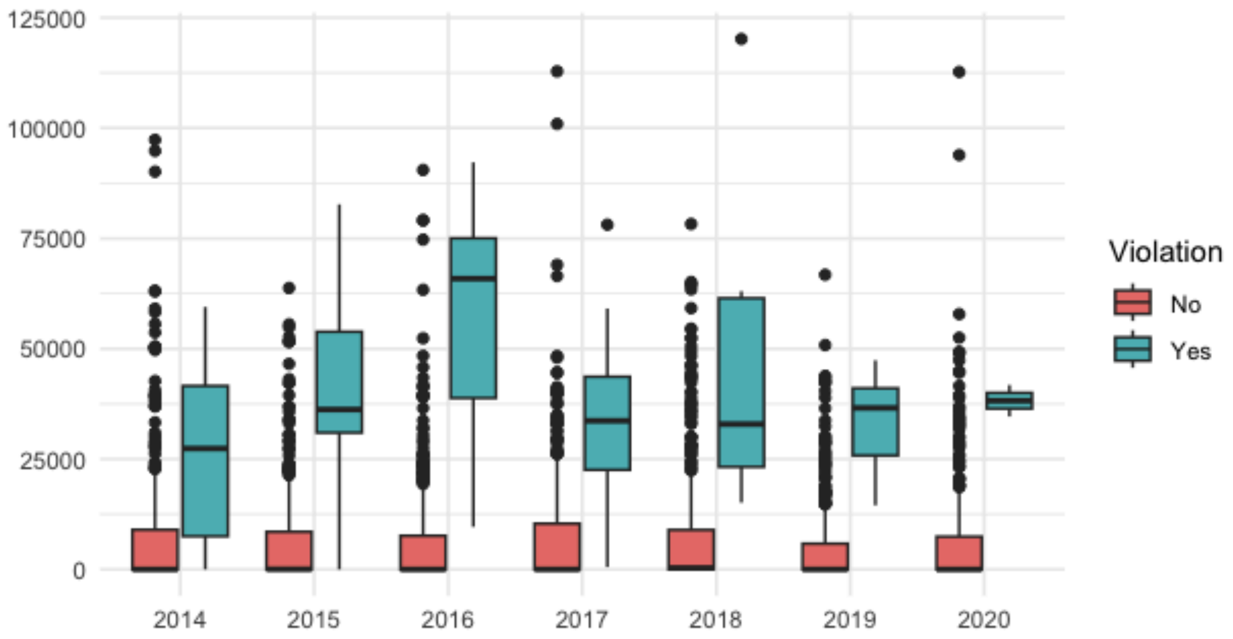

Supplementary Table 3. Likelihood of retention over time, by presence of a violation and magnitude of violation. Unit of analysis is the physician-guideline pair (eg., NPIs can be counted more than once). The year shown is the index year, which is the year assessed for whether a violation occurred. Panelists were defined as "retained" if they continued service on the NCCN Guidelines panel for at least 1 additional year beyond the index year (eg., for 2017, retention for the duration of 2018 was assessed, and panelists were grouped with respect to whether a violation had occurred in 2017). Panelists were also grouped with respect to the magnitude of the violation – less than or greater than 20% beyond the NCCN's stipulated limit (eg., less than or greater than $24,000 for the single-company limit, less than or greater than $60,000 for the all-companies limit).

**Year**	**2014**	**2015**	**2016**	**2017**	**2018**	**2019**	**2020**	**Total**
Physician-guideline pairs, total	483	499	507	531	528	521	554	**3623**
No violation	468	472	488	502	510	498	551	**3,489**
Number retained (%)	413 (88.2)	419 (88.8)	437 (89.5)	454 (90.4)	456 (89.4)	457 (91.8)	476 (86.4)	**3,112 (89.2)**
Violation, any magnitude	15	27	19	29	18	23	3	**134**
Number retained (%)	14 (93.3)	20 (74.1)	17 (89.5)	15 (51.7)	8 (44.4)	9 (39.1)	2 (66.7)	**85 (63.4)**
Violations that exceeded NCCN limit by <20%	8	12	10	7	8	5	2	**52**
Number retained (%)	8 (100)	9 (75.0)	10 (100)	5 (71.4)	4 (50.0)	2 (50.0)	2 (100)	**40 (76.9)**
Violations that exceeded NCCN limit by >=20%	7	15	9	22	10	18	1	**82**
Number retained (%)	6 (85.7)	11 (73.3)	7 (77.8)	10 (45.5)	4 (40.0)	7 (38.9)	0	**45 (54.9)**
